# Assessing Exposure to Unconventional Oil and Gas Development: Strengths, Challenges, and Implications for Epidemiologic Research

**DOI:** 10.1007/s40572-022-00358-4

**Published:** 2022-05-06

**Authors:** Nicole C. Deziel, Cassandra J. Clark, Joan A. Casey, Michelle L. Bell, Desiree L. Plata, James E. Saiers

**Affiliations:** 1 Department of Environmental Health Sciences, Yale School of Public Health, 60 College St., New Haven, CT 06510 USA; 2 Department of Environmental Health Sciences, Columbia University Mailman School of Public Health, 630 West 168th Street, Room 16-416, New York, NY 10032 USA; 3 Yale School of the Environment, 195 Prospect St., New Haven, CT 06511 USA; 4 Department of Civil and Environmental Engineering, Parsons Laboratory, Massachusetts Institute of Technology, 15 Vassar Street, Cambridge, MA 02139 USA

**Keywords:** Unconventional oil and gas, Exposure assessment, Children’s health, Epidemiologic studies

## Abstract

**Purpose of Review:**

Epidemiologic studies have observed elevated health risks in populations living near unconventional oil and gas development (UOGD). In this narrative review, we discuss strengths and limitations of UOG exposure assessment approaches used in or available for epidemiologic studies, emphasizing studies of children’s health outcomes.

**Recent Findings:**

Exposure assessment challenges include (1) numerous potential stressors with distinct spatiotemporal patterns, (2) critical exposure windows that cover long periods and occur in the past, and (3) limited existing monitoring data coupled with the resource-intensiveness of collecting new exposure measurements to capture spatiotemporal variation. All epidemiologic studies used proximity-based models for exposure assessment as opposed to surveys, biomonitoring, or environmental measurements. Nearly all studies used aggregate (rather than pathway-specific) models, which are useful surrogates for the complex mix of potential hazards.

**Summary:**

Simple and less-specific exposure assessment approaches have benefits in terms of scalability, interpretability, and relevance to specific policy initiatives such as set-back distances. More detailed and specific models and metrics, including dispersion methods and stressor-specific models, could reduce exposure misclassification, illuminate underlying exposure pathways, and inform emission control and exposure mitigation strategies. While less practical in a large population, collection of multi-media environmental and biological exposure measurements would be feasible in cohort subsets. Such assessments are well-suited to provide insights into the presence and magnitude of exposures to UOG-related stressors in relation to spatial surrogates and to better elucidate the plausibility of observed effects in both children and adults.

## Introduction

Unconventional oil and gas development (UOGD) involves the production of oil and natural gas from deep geologic formations such as shale using horizontal drilling and high-volume hydraulic fracturing. Shale gas production in the USA increased more than eightfold over the past decade, from 3,110 to 25,226 billion cubic feet from 2009 to 2019 [1]. In 2019, there were approximately 150,000 active UOG wells in the USA [2], with an estimated 1.5 to 4 million people living within 1.6 km of a UOG well [3]. Considerable community concerns and public debate have accompanied the rapid growth of the UOG industry regarding the multitude of potential human health hazards, including air pollution, noise, odors, water contamination, radioactive releases, seismic activity, traffic, and psychosocial stress [4, 5]. Potential benefits of UOGD include increased employment opportunities and wages, increased income for landowners who lease their land, lower energy prices, and domestic energy independence [6]. However, benefits are not distributed equally, may be short-term, and should be considered in the context of local and global environmental health impacts from fossil fuel extraction [7].

Epidemiologic studies have examined the human health impacts of UOGD, with the majority focused on children’s health [8]. While the bulk of these studies provide evidence of increased risk of adverse health outcomes in children (e.g., low birth weight, preterm birth, congenital anomalies, childhood asthma hospitalizations, and childhood cancer), studies have not been entirely consistent across endpoints. Limitations in exposure assessment methods can contribute to uncertainty about associations, and heterogeneity in methods can lead to different conclusions. In this narrative review, we (i) discuss the process and hazards of UOGD, (ii) describe the strengths, limitations, and challenges of environmental exposure assessment approaches relevant for UOGD health studies, and (iii) review the application of such methods in the epidemiologic literature, with a focus on studies of pediatric populations.

## Process of Unconventional Oil and Gas Development (UOGD)

UOGD, considered upstream oil and gas activity (versus downstream transport and refinement processes), is a complex process with multiple phases that vary over time and location [9]. The process starts with application and approval of the necessary permits. Next, the UOG well pad is prepared, which involves building access roads and clearing land where infrastructure for one or more UOG wells is stationed. Next is the drilling phase in which a borehole is drilled vertically approximately 1.6 to 3.2 km (1–2 mi) below ground, before the trajectory of the drill bit is turned to bore horizontally through the hydrocarbon-bearing rock layers. Steel pipes (casing) and cement are installed to maintain the integrity of the borehole and to isolate the upper portions of the borehole from the surrounding aquifer. Hydraulic fracturing then occurs, during which millions of gallons of water, chemical additives, and sand are injected into the well under high pressure and expelled through perforations in the casing to create fractures in the rock. When the pressure is released, the oil and/or gas, together with a portion of the injected fracturing fluids (flowback), move from the newly created fractures into the well and up to the surface for collection [10]. The production phase begins as oil and gas that flows up the well is separated at the surface from formation fluids (produced water) and transmitted through gathering pipelines from the wellhead to a storage facility or a processing plant. The approximately 7 to 14 million gallons of wastewater (flowback and produced water) generated during the operating lifetime of an UOG well [11] may be discharged to surface water following treatment, injected in class II injection wells, reused, or disposed of in unlined percolation pits, practices that vary by state [12]. Solid waste (e.g., drill cuttings) is commonly disposed of in municipal landfills [13]. At the end its production life, the well should be permanently sealed, although some wells are kept idle or abandoned and become increasingly likely to lose integrity as they age [14].

## UOG-Related Stressors

### Chemical Contaminants in Water

With more than 9 million people in the USA relying on drinking-water sources located within 1.6 km (1 mile) from a UOG well [9], water contamination from UOGD has been a major community concern [15, 16]. Hundreds of chemicals have been reportedly used in injection water or detected in wastewater, including known and suspected endocrine disruptors and carcinogens, such as metals, volatile organic compounds, polycyclic aromatic hydrocarbons, phthalates, and per- and poly-fluoroalkyl substances (PFAS) [9, 17–23]. Water contamination may occur due to surface spills of fracturing or wastewater fluids, release of improperly treated wastewater, structural failures, and well leaks [9, 12, 24–29]. Groundwater monitoring studies conducted thus far do not support widespread contamination [30–33]. However, groundwater and surface water impairments, spills, and violations have been documented across multiple states [15, 24, 26, 34–38].

### Air Pollutants

UOGD generates air pollutants from various sources, including well and road construction; use of diesel-powered construction, drilling, and transportation equipment and vehicles; dust generation during drilling; intentional flaring of natural gas; and volatilization of wastewater constituents [39–41]. Emission sources have different temporal profiles and may be continuous over varying time frames (e.g., diesel equipment, leaks) or be intermittent (e.g., flaring, venting). They may also occur at the well pad or off-site from transportation or associated UOG infrastructure [42]. Studies of airborne emissions or concentrations have identified several pollutants associated with UOGD activities, including carcinogens (e.g., diesel particulate matter, polycyclic aromatic hydrocarbons), and reproductive and/or developmental toxicants (e.g., ethylbenzene, toluene) [18, 39, 43–47].

### Radiation, Radioactivity, and Radon

Technologically enhanced naturally occurring radioactive compounds (TENORMS) have been detected in a variety of wastes generated by UOGD, including produced water [48–50], lateral drill cuttings [50], and impoundment sediments [51], and co-occur with sludges and mineral scales that accumulate inside UOGD equipment [48]. TENORMS have also been found in streambed sediments next to facilities managing UOGD wastewaters [52]. Additionally, higher indoor radon levels were observed in homes with more or closer UOG wells compared to those with fewer or farther UOG wells [53, 54]. Health effects of radiation exposure include adult and childhood cancers [55], impaired lung function [56], increased blood pressure [57], and oxidative stress [58].

### Sensory Stressors

Sensory stressors include noise, artificial light at night, and odors [59–62]. Noise pollution can activate the sympathetic nervous system; lead to annoyance, stress, and sleep disturbance; and potentially contribute to cardiovascular disease and adverse birth outcomes [62, 63]. Odorous compounds can reflect mixtures of volatile organic or sulfuric compounds, which have neurotoxic and respiratory effects, and can also trigger annoyance, concern, and anxiety at levels below established toxicity thresholds [64, 65].

### Socio-Environmental Disturbances

Social and environmental disturbances include, but are not limited to, deterioration of roadway infrastructure [66], increased traffic accidents [67], visible changes to the ecological landscape [68], and earthquakes [69]. Social impacts such as disruption of community cohesion [70], increased crime [71, 72], and decreased property values [73, 74] also threaten communities near UOGD.

### Greenhouse Gases

Methane, a potent greenhouse gas, is emitted to the air throughout UOGD from leaks and intentional releases (e.g., venting) [75]. Although methane is not directly toxic to nearby communities, except at very high levels in tap water, at which it poses a flammability danger, its indirect effects on future health risks through its role in climate change is an important threat to children’s health. This aspect is not generally considered in the current UOGD-related epidemiologic literature, which tends to be retrospective.

## Exposure Assessment Methods and Review of Epidemiologic Studies

**Fig. 1:**
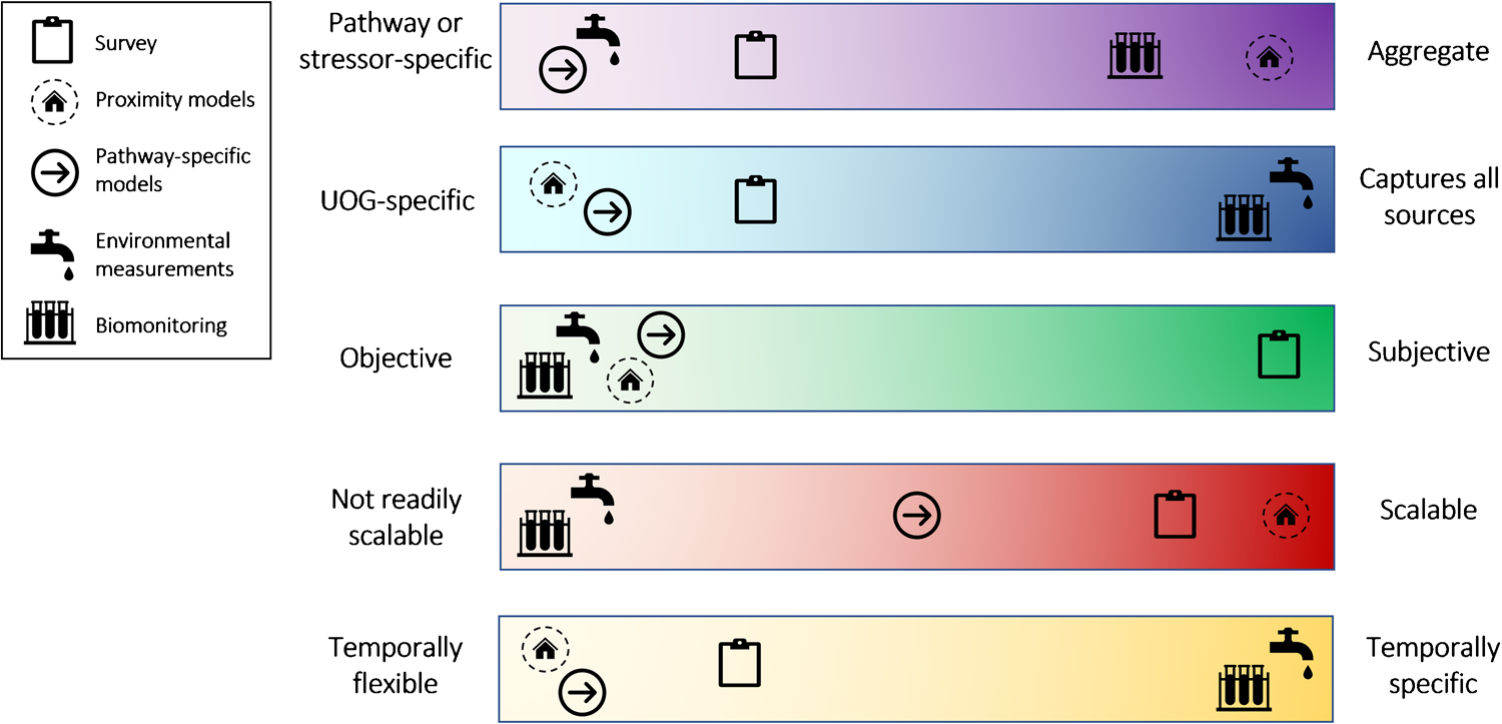
Characteristics of unconventional oil and gas development exposure assessment methods. The location of the icon indicates where each method lies on a spectrum of the respective characteristics

We review the features, strengths, and limitations of five categories of methods for assessing exposure to UOGD-related stressors [76]: surveys, environmental measurements, aggregate proximity-based models, pathway-specific models, and biological monitoring (Table 1, Fig. 1). We identified published epidemiologic studies designed to assess an exposure–response relationship between UOGD (or both conventional and UOGD) and human health conducted in the USA and Canada through searches in PubMed and Google Scholar using keywords related to oil and gas and health outcomes and reference lists of identified studies, with the last search conducted on January 31, 2022. While of public health value, we excluded small community surveys lacking clear comparison groups and studies of physical outcomes such as injuries or traffic accidents. Our search yielded 42 epidemiologic studies, of which 29 studies included a pediatric population or results related to children’s health (Table 2). We describe the exposure assessment methods used in each publication (Table 2).

**Table 1:** Strengths and limitations of UOGD exposure assessment methods for use in health studies

Exposure assessment method	Summary	Strengths	Limitations
Survey methods	Questionnaires, surveys, or interviews may be used to collect individual-level exposure information	Comparatively inexpensive Can be administered to many participants Can gather information on a wide variety of exposures and exposure-related factors Can ask about present and past behaviors and exposures Can gauge participants’ perceptions of exposure	Cannot capture exposures that are not known or observable to participants (e.g., chemical stressors) with precision or specificity Provides mainly qualitative information Information provided by participants may be subject to bias (e.g., recall bias), and retrospective information may be inaccurate
Aggregate proximity-based models and metrics	Distance and density-based mathematical models that assume exposure to UOGD varies in a predictable and consistent way in relation to proximity to source	Easily scalable for large studies Comparatively inexpensive Provides aggregate measure of exposure when etiologic agent is not known Often use publicly available information Can be used retrospectively or to align with etiologic windows of interest	Cannot separate individual etiologic agents or routes of exposure May not capture all routes of exposure (e.g., water) well or equally Based upon the assumption that UOG exposure varies in a predictable and consistent way in relation to proximity to source Does not address differences in exposures due to behaviors and activities May not reflect true patterns of environmental stressors (e.g., hydrologic patterns in comparison to buffer distance)
Pathway-specific models and metrics	Models designed to capture one or more specific exposure pathways (e.g., flaring, air pollution, radioactive material)	Scalable for large studies Comparatively inexpensive May provide more precise estimates than general spatial models when etiologic agent or pathway of concern is known Often use publicly available information Can be used retrospectively or to align with etiologic windows of interest	Generally requires large amounts of specific data that may not be available in all locations Computationally intensive Models have varying sources of error to account for (e.g., for the flaring model, double-counting of edge pixels in satellite imagery)
Environmental measurements	Quantification of UOG-related chemicals, elements, or compounds in air, water, soil, or other environmental media. Includes area monitoring and direct personal monitoring	Provides information on both location and magnitude of exposure Provides objective and quantitative measurements of specific etiologic agents Measurements may be incorporated into models and used to validate or improve models Repeated measurements may be used to quantify duration of exposure and temporal variations	Expensive and logistically difficult for large-scale studies (e.g., equipment, cost/comprehensiveness tradeoffs, strict protocols when interacting with human subjects) Chemicals used or produced not all known Nearly impossible to capture the numerous potential contaminants across the various media Timing sampling to coincide with intermittent or localized exposures (e.g., spills, release of air pollutants during peak production) is difficult and relies on timely and publicly available reporting of incidents Sampling at one point in time is unlikely to accurately represent long-term exposures Environmental measurements may not be reflective of individual exposure
Biological monitoring	Measurements of UOG-related chemicals, elements, or compounds in biologic media (e.g., urine, blood, hair)	Provides integrated measure of exposure from multiple pathways Accurate measure of individual-level exposure	Collection of biosamples requires participant cooperation and consent, strict adherence to ethical guidelines and protocols, and sample collectors trained in the necessary techniques (e.g., phlebotomy) Expensive Etiologic agent(s) must be known, and the large range of UOG-related compounds makes targeted biomonitoring challenging Biomarkers are not available for all contaminants Etiologic agents have varying half-lives, and a one-time measurement may not reflect long-term exposure Does not provide information on the source or route of exposure

**Table 2:** Features and applications of UOG exposure assessment methods in studies of UOGD and health outcomes

UOG exposure assessment method^a^	Description of exposure assessment	Lead author (year) by study endpoints			
		Adverse perinatal outcomes (*n* = 21)	Hospitalizations, asthma, cardiovascular diseases, or mortality (*n* = 12)	Cancer (*n* = 3)	Other health outcomes^b^ (*n* = 6)
Aggregate proximity-based metrics and models					
Presence or number of wells or permits per zip code, county, or other geographic unit	Area-based count density; number of wells per a specified area or administrative boundary	Ma (2016)^*^ [119] Tran (2021)^*^ [120] Busby (2017)^*^ [121] Tang (2021)^*^ [122] Tran (2020)^*,c^ [81•] Apergis (2019)^*^ [123] Hill (2018)^*^ [124] Hill (2022)* [79]	Jemielita (2015)^*^ [125] Willis (2018)^*^ [126] Denham (2019)^*^ [127] Denham (2021) [128] Willis (2020)^*,c^ [99] Peng (2018)^*^ [129] Johnston (2021) [130]	Fryzek [131] (2013)^*^ Finkel [132] (2016)	Deziel (2018) [133] Beleche (2018) [134] Komarek (2017) [72] Johnson (2020) [135]
Distance to UOG well	Geographic distance between residence and nearest UOG well	Currie (2017)^*^ [136] Willis (2021)^*^ [137•]	Koehler (2018)^c^ [42]		
Inverse distance weighted well count	A count of all wells within a prescribed area around a home (i.e., buffer) that gives more weight to wells located closer. May be intensity-adjusted or phase-specific	Stacy (2015)^*^ [138] McKenzie (2014)^*^ [139] Whitworth (2018)^*^ [82] Whitworth (2017)^*^ [140] Janitz (2019)^*^ [141] Caron-Beaudoin (2021)^*^ [142]	Koehler (2018)^c^ [42] Li (2022) [98]	McKenzie (2017)^*^ [143]	
Inverse distance-squared weighted	A count of all wells within a prescribed area around a home (i.e., buffer) that gives more weight to wells located closer. Assumes that the exposure potential declines rapidly with distance, as a function of the distance squared	González (2020)^*^ [83]			
Activity-based metric	A count of all wells within a prescribed area around a home that gives more weight to wells located closer. Assumes that the exposure potential declines rapidly with distance, as a function of the distance squared. Assumes that each phase of well development has different exposure potential that may be represented by features of the well (e.g., total well depth, daily natural gas production volume)	Casey (2016)^*^ [80] Casey (2019)^*^ [144] Tran (2020)^*,c^ [81•]	McAlexander (2020) [145] Koehler (2018) [42]^c^ Rasmussen (2016)^*^ [104] Willis (2020)^*,c^ [25]		Tustin (2016) [146] Casey (2018) [147]
Pathway-specific models and metrics					
Air pathway		McKenzie (2019a)^*^ [96•]	McKenzie (2019b) [97] Li (2022) [98]		
Flaring		Cushing [84•] (2020)^*^	Willis (2020) [99]^*,c^		

^*^Indicates a study with a pediatric population or a population that includes both children and adults with results presented separately for children

^a^Note that no epidemiologic studies included in this review used surveys, environmental measurements, or biological monitoring to assess exposure to UOGD

^b^Other health outcomes includes studies of self-reported health symptoms, mental health outcomes, nasal/sinus symptoms, fatigue, migraine, and sexually transmitted infections

^c^This study applied multiple metrics that fit into multiple categories

## Survey Methods

### Overview

Surveys are used to gather self-reported, individual-level information on a wide variety of exposure-related factors, such as participant behavior, activities, observations, and perception of UOGD. None of the epidemiologic studies identified in this review utilized this method (Table 2).

### Strengths

Surveys are comparatively inexpensive tools that can be administered to many individuals across a vast geographic scale and allow for acquisition of current and historical information. Surveys have been useful for assessing perceived exposures such as the felt experience of earthquakes, which may be as or more relevant for health outcomes as compared to objective measures of seismicity. For example, the US Geological Survey (USGS) Did You Feel It (DYFI) is a crowd-sourced database where individuals submit an online report to the USGS about the time, location, and felt impact of an earthquake [77]. USGS uses these reports to estimate a community decimal intensity for each seismic event at the ZIP code level. In addition, participant self-reported housing damage has shown strong correlation with cumulative ground motion assessed from shakemaps (i.e., visual displays of ground shaking occurring at different locations immediately after an earthquake). In terms of UOGD, residents have also reported on observable changes to their air or water, such as odors, change in the color, appearance, turbidity, or foaming of their drinking water [16, 30, 78].

### Limitations and Challenges

The utility of exposure assessment surveys is limited to hazards that are directly knowable and observable by residents and therefore are not suitable for most UOGD-related chemicals and radioactive materials, which are odorless, colorless, and not detectable by human senses. Moreover, even observable changes (e.g., color change, foaming, turbidity, odor) cannot readily be translated into quantitative exposure estimates for specific agents.

## Models and Metrics

### Overview

Human exposure models are mathematical expressions of attributes of the environment, community, or individual that are expected to influence exposure. These can take several forms, such as geostatistical models that map proximity to a contaminant source or physically based models that draw upon established principles governing how contaminants move through various media (e.g., air, water, soil) to predict the transport of a stressor from source to receptor. Here, we group models into aggregate proximity-based models, which treat UOGD as a collective entity, and pathway-specific models which focus on a particular source or exposure pathway related to UOGD. All epidemiologic studies published to date have used models to assess exposure to UOGD, with the majority using aggregate approaches (Table 2).

## Aggregate Proximity-Based Models and Metrics

### Counts or Geographic Aggregation

These metrics consider presence or number of UOGD facilities per ZIP code, county, or circular buffer around a residence, used in 21 epidemiologic studies to date (Table 2). This metric assumes that residents of geographic units with higher numbers of UOGD facilities experience higher levels of exposure. Because environmental exposures do not respect administrative boundaries, use of these types of spatial units is subject to edge effects (i.e., wells located just across a Census tract border). These metrics have recently been extended to estimate counts of UOG wells around other receptors, such as drinking water sources [79].

### Distance

The mapped Euclidean distance to the UOG well nearest to a home or other receptor is another simple metric that assumes that homes closer to UOG wells are more likely to be exposed or face higher levels of exposure. This calculation is based upon a single well—the closest well—and does not incorporate the density of wells in the area surrounding a residence.

### Distance-weighted metrics

Inverse-distance or inverse distance-squared weighted UOG well counts improve upon simple distance measures by accounting for the number of UOG wells within a buffer zone (generally 1, 2, 5, or 10 km) while weighting the closer wells more heavily than the distant wells by assuming a decline in exposure potential as an inverse (Eq. 1) or inverse-squared (Eq. 2) function of distance.

1 $$\mathrm{IDW}\:\mathrm{well}\:\mathrm{count}=\sum\nolimits_{\mathrm i=1}^{\mathrm n}\frac1{{\mathrm d}_{\mathrm i}}$$

2 $$\mathrm{ID}^2\mathrm W\:\mathrm{well}\:\mathrm{count}=\sum\nolimits_{\mathrm i=1}^{\mathrm n}\frac1{\mathrm d_{\mathrm i}^2}$$

where *d* is the distance between the *i*th UOG well and a residence, and *n* the number of UOG wells.

These models assume all wells have the same maximum exposure potential regardless of production volume, wastewater produced, well phase, topography, or meteorology. Distance-weighted metrics have been commonly used in epidemiologic analyses (*n* = 9 studies, Table 2).

### Activity-Based Metrics

Activity-based models extend distance-based metric by also including attributes of UOG wells expected to influence exposure potential, such as vertical well depth or production volume [41, 80, 81•]. Nine health studies to date have used some form of an activity-based metric (Table 2).

### Strengths

Aggregate proximity-based models offer the advantage of being scalable (i.e., feasible to apply to large geographic areas or populations) and thus practicable for assignment of exposure to thousands of wells across the thousands of subjects sometimes included in health studies. Proximity-based spatial surrogates offer the opportunity to assess exposures retrospectively, which is important if the etiologically relevant time windows occur in the past. They can also be calculated to align windows of health vulnerability (e.g., trimesters) with the phases of UOGD to create phase-specific metrics (e.g., UOG exposure during production). These temporal considerations are of particular importance for adverse birth outcomes and childhood cancer, because effect estimates can vary based on the timing of exposure [82, 83]. Conversely, other studies of adverse birth outcomes have observed strong correlations in exposure assessments across trimesters, making it difficult to examine potential differences [84•, 85].

These approaches also serve as an aggregate measure of the myriad of physical, chemical, and social stressors potentially associated with UOGD. This aggregation is a useful feature for this complex industrial process, where exposures to multiple hazards are likely and the dominant stressor is not known and may differ from well to well. Additionally, the linkage of health data or risks with relatively simple metrics like distance between home and UOG well is directly relevant and actionable for policy makers and stakeholders; for example, such data can be used to inform the establishment of setback distances, the minimum allowable distance between an oil and gas well and sensitive human receptors (e.g., residences, schools, or hospitals).

### Limitations and Challenges

Proximity-based spatial surrogates do not allow for identification of etiologic agents underlying observed associations [86]. Additionally, the effects of processes affecting chemical fate and transport, such as advection, dispersion, and transformation reactions that alter contaminant toxicity, are unlikely to be approximated by simple spatial metrics. Moreover, the accuracy of the underlying assumption that UOG-related exposures diminish predictably with distance likely varies by exposure type. For example, the impacts of increased traffic, ecological disturbance, or formation of secondary air pollutants may not be appropriately captured by a model that presumes a linear attenuation of exposure intensity with increasing distance. Approaches that are based on buffers (e.g., 1 km radius around a residence) are limited by the fact that the actual exposures do not follow perfect circular patterns. Because these metrics have only been compared to environmental measurements in a small number of studies (e.g., 30, 41, 94), our understanding of their ability to capture specific etiologic agents is still unfolding.

The epidemiologic studies conducted to date have predominantly relied on secondary data sources, such as birth, hospitalization, or electronic medical records, without participant contact. This has necessitated the use of single address (e.g., maternal residence at birth) to assign exposures, which could introduce exposure misclassification due to unaccounted residential mobility [87, 88]. While residential mobility is an important consideration in studies of children’s health, risk estimates have generally been robust to the impact of residential mobility on exposure assessments [89, 90]. The potential to introduce error is compounded if evaluating diseases with longer exposure windows or latency periods (e.g., adult cancer).

## Pathway-Specific Proximity-Based Models and Metrics

### Overview

In contrast to aggregate models, approaches have been developed to capture specific stressors such as air emissions [41], flaring [91], drinking water vulnerability [92], radioactivity [93•], earthquakes [94, 95], and ancillary UOG infrastructure [42]. The strengths and limitations for these approaches are discussed below. To date, five epidemiologic studies have applied a pathway-specific exposure model (Table 2).

### Air Pathway Models

An activity model included weighting factors based on air emissions data that accounts for changes in emissions between and within phases of well development was constructed for Colorado [41] and subsequently applied in an epidemiologic study of congenital malformations and adult cardiovascular endpoints [96•, 97]. Limitations include the requirement of specific UOG data (e.g., production volumes and number of tanks per well pad) that may not be readily available in all states. Application to other regions would require assumptions that the emissions are correlated with the measured factors and that these are similar across producers and formations. In addition, while targeting the air pathway, it does not provide information on specific pollutants of concern.

Li et al. (2020) constructed a downwind proximity-based model and compared it to radioactivity measurements available from a national network of environmental radiation monitors [93•]. They created circular sectional buffer around each residence. To isolate emissions from upwind industrial activities, they sliced a 90° circular sector centered on the daily upwind direction for each circular buffer and summed the number of UOGD wells in each wedge-shaped buffer based on daily wind fields. They found an association between UOGD wells and downwind radiation measurements. This downwind model was applied in one health study of all-cause mortality among Medicare beneficiaries, which observed significant higher mortality risk associated with living downwind of UOGD wells [98].

### Flaring-Specific Model

A flaring-specific model was developed for Texas using data from the Visible Infrared Imaging Spectroradiometer (VIIRS) Nightfire satellite instrument (which detects thermal sources) alongside reported estimates of vented and flared gas from the Railroad Commission of Texas [91]. The model was used to identify flaring events and to estimate the gas volume of each flare in a study of birth outcomes in Texas, which found that exposure to a higher number of flaring events was associated with increased risk of preterm birth [84•]. This model provides high resolution estimates of flared locations and volumes using publicly available VIIRS data, which covers the entire planet (albeit asynchronously). The model may overestimate the number of flares in a region due to artifacts of VIIRS [91]. Willis et al. (2020) used flaring volumes by zip code data to classify exposures (among other metrics) in a study of pediatric asthma hospitalizations in Texas [99]. While there was an association between oil and gas well density and increased hospitalizations, the results specific to flaring volumes were inconclusive.

### Metrics for Injection-Induced Earthquakes

Exposure to earthquakes can be based on location of the epicenter, timing of the earthquake, and magnitude of the seismic event. Generally, earthquakes greater than (or equal to) magnitude 4 have been considered relevant for human exposure and health. These have been used to assess short-term exposure windows for acute health outcomes, such as 1 month prior to health events [94], and longer-term windows such as summing cumulative peak ground acceleration from shakemap data over a 6-year period [95]. Prior work has connected earthquakes to adverse perinatal and child health [100–103], but no studies have evaluated the role of UOGD-induced earthquakes and adverse birth outcomes.

### Ancillary UOGD Infrastructure

Few studies have considered UOGD infrastructure beyond the well pad. Koehler et al. (2018) incorporated compressor station locations into an inverse-distance squared exposure metric that also included pad development, drilling, hydraulic fracturing, and gas production [42]. This metric performed similarly to a previously used inverse distance squared metric that did not incorporate compressor stations in evaluations of respiratory outcomes [104]. The authors hypothesized this occurred because compressor stations were co-located in space and time with the other phases of UOGD activity measured.

### Hydrological Models and Metrics

Physics-based estimates of water-well vulnerability (i.e., the likelihood of contamination) that draw on hydrological principles to simulate groundwater flow rates and patterns can improve exposure assignments in human-health studies focusing on the drinking-water pathway. Soriano et al. (2020) used hydrologic models to simulate the capture zones of residential drinking-water wells and estimated vulnerability based on the probability that a capture zone encompassed UOG infrastructure (i.e., well pads) [105]. Physics-based vulnerability estimates can also predict the likelihood of future exposures, thereby enabling households of greatest risk to be targeted for prospective monitoring and preventative actions to minimize exposure to UOG contaminants. Physics-based models can be computationally intensive and are challenging to scale to large geographic areas required of many population-based epidemiologic studies. To circumvent this challenge, Soriano et al. (2021) used vulnerability estimates of a physics-based model of groundwater flow and chemical transport to train a machine-learning model that classified the vulnerability of household wells on the basis of predictors readily computable from a geographic information system [92]. A predictor combining information on topography, hydrology, and proximity to contaminant sources (inverse distance to nearest upgradient UOG source) was found to be highly important for accurate machine-learning model predictions of vulnerability. This new metric was recently applied as an exposure indicator in a groundwater monitoring study by Clark et al. (2022) [106•] and has not yet been applied in any published epidemiologic studies.

### Strengths

Pathway-specific models may offer improved accuracy over simple proximity models due to their incorporation of chemical transport, source characteristics, land use, meteorology, and other factors. This can reduce exposure misclassification in epidemiologic studies and help disentangle the effects of different exposure routes or hazards, providing important information for additional monitoring studies or exposure mitigation strategies.

### Limitations and Challenges

Pathway-specific models may be computationally intensive and require substantial information for parameterization that may be difficult to acquire. The increased complexity can make the models less readily scalable to large geographical areas, although recent advances in computational and statistical approaches are helping to circumvent this issue. In addition, more complex models may be less readily interpretable to the public and policy makers. If the models predict exposures at a single residential address, they are prone to the same error as the simple proximity models in terms of residential mobility.

## Environmental Measurements

### Overview

Environmental measurements are quantitative measurements of samples collected from various media (e.g., air, water, soil, and dust) that can be used to calculate exposure. Environmental measurements can be collected directly at the point of contact (e.g., a personal air monitor worn by an individual) or from area or ambient settings (e.g., a stationary air monitor in a home). No large-scale epidemiologic analyses have used environmental measurements to assess UOG exposure to date (Table 2).

### Strengths

Measurement of targeted analytes often has standard methods and can be robustly quantitative. Repeated measurements could help quantify duration and temporal variations in exposures. Measurements can be incorporated into models as weighting factors or can be used to evaluate or calibrate models. Measurements can be used to establish exposure pathways or assess the dominant pathways, demonstrating whether agents reportedly used in UOG development are reaching human receptors and by which mechanisms. Environmental measurements can be highly relevant to individual exposure and can be reported to stakeholders and residents. These can also be compared to health-based standards and guidelines where available. Untargeted analyses can reveal the presence of unexpected contaminants.

### Limitations and Challenges

Measurements (particularly those with sufficient spatiotemporal resolution to be informative at an individual level) may not be feasible or may be prohibitively costly in a large-scale epidemiologic study. Furthermore, monitoring networks do not exist for many relevant environmental stressors. Given the lack of timely and publicly available reporting of UOG-related incidents (e.g., spills, leaks) and the resource-intensive nature of environmental sampling, it is difficult to coordinate sampling efforts with environmental releases. As a result, if monitoring is conducted at only a single time point, it is unlikely to represent the full range of past exposures or peak exposure at that location. This issue is particularly salient for groundwater measurements given the lack of monitoring and regulatory oversight of domestic groundwater wells [104, 107] which may be shallow and made even more vulnerable to contamination as their casing and cement seals deteriorate with age. Monitoring is further complicated by the challenging and non-random access to such groundwater (i.e., permission must be granted by a property owner) [105, 108]. For both air and water, it is impossible to measure everything of interest because there are numerous potential pollutants of public health concern [17, 18]. Environmental sampling also requires carefully designed protocols, participant cooperation and consent, quality control procedures, and appropriate analytical capabilities and materials to properly collect and quantify target analytes.

Identification of target analytes for measurement is challenging because constituents of fracturing fluids are reported in an inconsistent and voluntary manner [23, 109, 110]. Naturally occurring compounds mobilized by the UOG process and transformation products that are not used deliberately may evade targeting for analysis [111, 112]. There are also limited data for comparison, although there are efforts to build publicly available datasets of detections to complement disclosure databases [113]. In terms of water measurements, publicly available pre-drill data are sparse [33, 114], and many contaminants of emerging concern do not have health standards or guidelines. As such, it is difficult to identify exposures that are “elevated” or present a definitive health risk. For air pollution, there are fewer federal monitoring stations in rural areas that tend to host UOGD compared to heavily populated metropolitan areas, and a small number of atmospheric pollutants are systematically measured.

Finally, because true exposure is a combination of environmental concentrations and human behaviors and activities, collected measurements may not be representative of actual human exposure or reflect heterogeneity in exposures across persons. Individuals are rarely stationary, and their exposure profile will be highly influenced by the different microenvironments where they spend time as well as their behavior and activities in those microenvironments. Direct personal air sampling devices can overcome this issue, or surveys can complement measurements with individual-level information about behaviors and activities. For example, with respect to water-based exposures, people may consume water or other beverages from multiple locations or may have treatment systems that alter the water chemistry. A study of 255 homes in Pennsylvania and Ohio located in areas with UOGD found that more than 20% of their participants reported drinking primarily bottled water, which could come from many sources, instead of water from their private well [106•].

## Biological Monitoring of Exposure

### Overview

Biological monitoring is a direct measure of chemical exposure that integrates all potential routes of exposure into one measurement of internal dose. No large-scale epidemiologic studies have used biological monitoring to assess UOG exposure (Table 2), and to our knowledge, only two pilot studies have measured exposure biomarkers among individuals living in a community with UOG activity [115, 116]. These studies observed elevated levels of benzene metabolites and manganese in urine and manganese, barium, aluminum, and strontium in the hair of pregnant women living atop the Montney Formation in Northeastern British Columbia, Canada, as compared to reference populations in both Canada and France. Individual measurements were not compared to proximity to or density of UOG activity, meaning it is possible the population was exposed to other sources of VOCs and trace metals.

### Strengths

Biological measurements provide an integrated and direct measure of total body burden that reflect multiple exposure pathways and routes and the different activities and microenvironments of individuals. Biological monitoring may also be useful in identifying etiologic agents associated with health outcomes.

### Limitations and Challenges

The broad range of compounds associated with UOGD complicates targeted biomonitoring. Because UOG chemicals typically have numerous indoor and outdoor sources, biological monitoring cannot provide insights into the dominant source or route of exposure. Many biomarkers have short half-lives and therefore new measurements may not reflect prior exposures during etiologically relevant time windows. Finally, to collect biosamples, researchers must obtain participant consent, adhere to ethical guidelines and protocols, and train sample collectors in the necessary methodology (e.g., phlebotomy), all of which raise cost and prevent barriers to broad, systematic study. Although children often have a higher body burden of environmental chemicals compared to adults, collection of biospecimens may be particularly challenging or not possible among pediatric populations, for reasons of practicality (e.g., compliance, ability to achieve sufficient sample volume) and ethics (e.g., the invasive nature, obtaining informed assent) [117, 118].

## Conclusions

In this review, we describe the features of UOG exposure assessment methods, which are summarized in Fig. 1. We also enumerate the many challenges facing UOGD exposure assessment, including limited information on the identity and intensity of chemical and non-chemical stressors, sparse monitoring data in areas with active UOGD, the episodic or infrequent release of chemicals or other hazards, and the complexity of the different stressors with regard to temporal, spatial, release, and dispersion patterns. Despite these issues, published epidemiologic studies generally relying on relatively simple, aggregate proximity-based models, have consistently identified increased risk of health problems, particularly in children. Improved methods could potentially reduce uncertainty and provide evidence for specific exposure pathways. While the substantial analytic capabilities required for large-scale environmental measurements may make them impractical for use in a traditional epidemiologic study, such studies are highly valuable and could shed light on pathways of exposure, particularly in a cohort subset. Not all stressors lend themselves to the approaches discussed in this review, such as estimating the health impacts of greenhouse gases (e.g., methane), which may not immediately or directly affect health but will hold global consequences for future generations. As epidemiologic investigation of UOGD is still a relatively nascent research area, UOGD exposure assessment methods have already progressed substantially and will continue to be refined as new information comes to light and researchers tackle these challenges.
